# Activity of Various Essential Oils Against Clinical Dermatophytes of *Microsporum* and *Trichophyton*

**DOI:** 10.3389/fcimb.2020.545913

**Published:** 2020-10-16

**Authors:** Nicole Parrish, Stefanie L. Fisher, Ashlea Gartling, David Craig, Nicholas Boire, Joshua Khuvis, Stefan Riedel, Sean Zhang

**Affiliations:** ^1^Department of Pathology, The Johns Hopkins University School of Medicine, The Johns Hopkins Medical Institutions, Baltimore, MD, United States; ^2^Beth Israel Deaconess Medical Center, Boston, MA, United States

**Keywords:** dermatophytes, essential oils, antifungal, synergy, mechanism

## Abstract

Dermatophytoses account for nearly a quarter of all fungal infections worldwide. These difficult to treat infections of the skin, hair, and nails, are growing more resistant to conventional antifungal treatments, and when treatable, often require prolonged therapeutic regimens. For centuries, essential oils have been used to treat a variety of ailments. In this study, we evaluated the clinical effects *in vitro* of 65 essential oils and 21 essential oil blends against various clinical species/strains of dermatophytes from two primary genera, *Microsporum* and *Trichophyton*. Our aim: To determine the overall activity of a wide range of essential oils against a number of clinical strains of dermatophytes. For all assays, 16 clinically derived species/strains of dermatophytes were used. The activity of each essential oil was assessed using a modified disk-diffusion assay over a period of 21 days of incubation vs. standard antifungal drugs. Subsequently, we determined the minimum inhibitory dilution possible for the most potent essential oils and performed combination testing to determine if synergy could be demonstrated with sub-inhibitory concentrations. We also assessed the effect of repeated vs. single applications. Of all the essential oils tested, cassia, cilantro, cinnamon, thyme, and oregano were the most potent along with one blend, DDR Prime; all genera/species tested were completely inhibited for 21 days following a single application. Many of the other oils tested exhibited temporal differences in activity where significant inhibition was observed ≤10 days of incubation which declined by day 21. Synergistic combinations were achieved with oregano and cilantro, cassia, or cinnamon bark; rose and cassia were also synergistic. Repeat application maintained complete inhibition for citronella, lemon myrtle, and litsea out to 21 days, but not lemon grass or On Guard. More study is necessary to understand the ways essential oils inhibit the growth of dermatophytes. Comprehensive research aimed at understanding the mechanism of action of essential oils and their components may provide the basis for a natural alternative to topical antifungal drugs. Such research could be envisioned to target optimal combinations and determine the timing between applications to provide for maximum inhibition of recurrence or growth.

## Introduction

Dermatophytoses account for 20–25% of fungal infections worldwide, with a prevalence approaching one billion as of 2010 (Havlickova et al., 2008). In 2004, it was estimated that there were over 29.4 million cases of cutaneous fungal infections in the US accounting for over 51 million physician visits (Ghannoum et al., 2000; Bickers et al., 2004; Suh et al., 2006; Gupta et al., 2017). Infections caused by dermatophytes affect the keratinized layers of the, skin, hair and nails and the causative agents belong to one of the following three genera: *Tricophyton, Microsporum*, or *Epidermophyton*. While most dermatophyte infections are not life-threatening and respond well to currently available topical treatment with over-the-counter (OTC) fungal agents, some dermatophyte infections can, however, can be difficult to treat, require prolonged therapeutic regimens, and are increasingly resistant to conventional antifungal therapies. Previous estimates put the total cost for treatment of dermatophyte infections in the US at $1.67 billion (Degreef and DeDoncker, 1994; Mukherjee et al., 2003; Santos and Hamdan, 2007; Gupta et al., 2017). In addition to costs associated with treatment, many currently used antifungal agents have significant side effects thus underscoring the need for identification of therapeutic alternatives including those from natural products such as plant-based bioactive compounds, essential oils, and/or their components (Gupta et al., 2016; Lopes et al., 2016). Essential oils are complex mixtures which typically consist of a variety of low molecular weight compounds which can range in number up to 100 or more with a select few being the most abundant (Raut and Karuppayil, 2014; Sharma and Malik, 2015; Lopes et al., 2016). The composition of essential oils can vary due to a number of factors including the extraction method used, the type and species of plant from which they are derived, the composition of the soil, and the exact stage of growth at the time of harvest. For this reason, it is important that careful chemical analyses be performed using methods such as gas chromatography-mass spectrometry (GC-MS) to verify and standardize the composition of essential oils to ensure batch to batch consistency over time. Previous investigators have evaluated the use of EO's against dermatophytes focusing largely on melaleuca, thyme, eucalyptus, oregano, and lavender (Zuzarte et al., 2009, 2011; Lopes et al., 2017). Additional studies have been conducted which investigated the antifungal effects of specific components of these and other EOs including mono-, di-, and sesquiterpenes, phenolic terpenes, phenylpropanoids, hydrocarbons, and other cyclic compounds (Tullio et al., 2007; Jantan et al., 2008; Miron et al., 2014a,b). Some investigations have led to the conclusion that the anti-dermatophytic activity resulted from synergy between major and minor components rather than the result of the presence of a single compound (Elaissi et al., 2012). In this regard, there is much to learn about these complex mixtures, including expansion of the number of EOs tested. In the current study, we evaluated a large number of EOs for which GC-MS data was available and determined their activity *in vitro* against *Trichosporum* and *Microsporum* species of clinical importance.

## Materials and Methods

### Determination of Essential Oil Activity Using a Modified, Disk-Diffusion Assay

A total of 65 individual essential oils and 21 blends (doTERRA®, Salt Lake City, Utah) were tested and are shown in Table 1. For all assays, a total of 16 clinically derived strains of each of the following genera/species were used: *Microsporum canis (n* = *2), M. gypseum (n* = *2), M. audouinii (n* = *1), Tricophyton tonsurans (n* = *2), T. mentagrophytes (n* = *2), T. rubrum (n* = *2), T. sudanense (n* = *2)*, and *T. violaceum* (*n* = 3). No isolates of *Epidermophyton* were available, so this genus was not included in the study. All isolates were cultured on Mueller Hinton agar plates (100 mm × 15 mm; Hardy Diagnostics, Santa Maria, CA) and incubated at 30°C until adequate sporulation was observed (Pinto et al., 2006). Spore suspensions were prepared in 2 ml of sterile water, vortexed for 1 min, and viable counts determined by plating 100 μl of 10-fold dilutions to Mueller Hinton agar plates (Hardy Diagnostics). Spore suspensions were subsequently used to plate fungal lawns on separate Mueller Hinton agar plates to which sterile blank filter disks (6 mm, Becton Dickinson, Sparks, MD) were added (Pinto et al., 2006). Individual essential oils or blends (Table 1) were added to each disk (10 μl/disk) and allowed to air dry for 10 min. Plates were incubated at 30°C for 21 days and examined daily for growth. Zones of inhibition were measured in mm over the 21 days and changes in zone diameter noted over time. Zone interpretations were placed into the following categories: complete inhibition (zone diameter 80.0 mm, equivalent to the diameter of the plate), near-complete inhibition (zone diameter 74.0–79.9 mm), minor to moderate inhibition (31.0–71.9 mm), and little to no inhibition (zone diameter <31 mm). Itraconazole (10 μg/ml, Sigma-Aldrich, St. Louis, MO) and terbinafine (30 μg/ml, Cambridge Reagents, East Yorkshire, UK) were used as a comparative reference for essential oil mediated zones of inhibition where a distribution curve for these two antifungal drugs was established. This distribution curve was then used to determine the range for each antifungal drug, known to vary in potency from high (terbinafine) to low (itraconazole) against various dermatophyte genera/species. Relative potency for each individual oil/blend was determined for each respective fungal strain tested by comparison to the range established for terbinafine and itraconazole alone.

**Table 1:** Single essential oils and blends used in this study.

**Single oils (*****n*** **=** **65)**		**Blends (*n* = 21)**
Amyris	Howood	Anti-aging
Arborvitae	Jasmine	Aroma touch
Austrian fir	Juniper berry	Balancing
Basil	Labdanum	Breathe
Bergamot	Lavandin	Citrus bliss
Black cumin seed	Lavender	Clary calm
Black pepper	Lemon	Clear skin
Blue chamomile	Lemongrass	DDR prime
Blue tansy	Lemon myrtle	Deep blue
Camphor	Lime	Digestion
Cardamom	Litsea	Elevation
Cassia	Mandarin	Focus
Catnip	Marjoram	On guard
Cedarwood	Melaleuca	Purity
Cilantro	Myrrh	Serenity
Cinnamon	Oregano	Slim-n-Sassy
Citronella	Osmanthus (absolute)	Tension
Clary Sage	Patchouli	Terra shield
Clementine	Peppermint	Topical
Clove	Roman chamomile	Whisper
Cocoa	Rose	Zendocrine
Coriander	Rosemary	
Cypress	Sandalwood	
Eucalyptus	Siberian fir	
Fennel	Tangerine	
Fennel (sweet)	Thyme	
Fir needle	Vanilla	
Frankincense	Vetiver	
Geranium	White Fir	
Ginger	Wild orange	
Grapefruit	Wintergreen	
H. Sandalwood	Ylang ylang	
Helichrysum		

### Determination of Minimum Inhibitory Concentrations (MIC's) for the Most Potent Essential Oils Using a Modified Agar Dilution Assay

Isolates were selected for MIC determination which ensured that differences in susceptibility between genera, species and strains were included. One isolate, *M. canis* #2, could not be tested with all of the oils due to a loss of viability. Essential oils for which MICs were determined included cinnamon bark, cassia, lemon grass, cilantro, litsea, citronella, osmanthus, rose, lemon myrtle, thyme, and oregano. Since each essential oil represented a complex mixture of varying numbers of individual compounds, determination of an MIC in the conventional sense was not possible. Thus, 2-fold dilutions (1:2–1:8) of each essential oil to be tested was prepared in fractionated coconut oil (doTERRA®), which was used as a biologically inert diluent, and 10 μl was added to 30 mls of Mueller-Hinton agar suspension cooled to 55°C. Control plates were made which incorporated the highest volume of fractionated coconut oil used (10 μl).

Once each essential oil had been mixed well with the 55°C agar suspension it was poured into individual petri dishes and allowed to solidify at room temperature in a biological safety cabinet. Subsequently, spore suspensions from each individual strain were made using 2 ml of sterile water, vortexed for 1 min, and 10-fold dilutions prepared ranging from 10^−1^ to 10^−5^ CFU/ml. Individual dilutions (100 μl) were inoculated onto control and essential oil-containing plates, distributed evenly over the agar surface using sterile glass beads, and followed by incubation at 30°C for 21 days. Viable counts on control plates vs. essential oil-containing plates were determined and the lowest dilution of oil resulting in ≥99% inhibition of fungal growth was defined as the MIC.

### Synergy Testing

Based on the MIC data, selected essential oils (cinnamon bark, cassia, cilantro, rose, and oregano) were used for synergy testing against 4 dermatophyte species: *T. mentagrophytes*, strain #1, *T. tonsurans*, strain #2, *T. rubrum*, strain #1, and *M. gypseum* strain #1. These strains were selected since each had an average zone of inhibition for all essential oils considered together of <30 mm or less than the range for the least inhibitory standard drug (itraconazole). Plates and suspensions were prepared as above for MIC testing except that initial stocks of essential oils to be incorporated into the agar were diluted in fractionated coconut oil at least 2-fold or greater (1:2, 1:4, 1:8 v/v) below the individual calculated MIC specific for each species/strain to be tested. Once initial essential oil dilutions had been made, combinations were prepared by mixing two or three of the diluted oils together (1:1, v/v) and adding 10 μl of each combination to 30 mls of Mueller-Hinton agar suspension cooled to 55°C. A corresponding control was used for each strain which incorporated the same volume of fractionated coconut oil (10 μl). Essential oil combinations were selected based on the predominant constituent(s) known to be present in each oil based on mass spectrometry data provided with the oils (data not shown, aldehydes: cilantro, cinnamon bark, and cassia; phenols, oregano; alcohols, rose). Grouping of individual oils by primary component(s) permitted various combinations to be tested where one oil from each representative group could be combined with others rather than multiple oils from the same group. Synergy was defined as combinations resulting in complete inhibition at 21 days of incubation vs. a complete lack of inhibition at the same time point for the same dilution of each essential oil alone.

### Effect of Repeat Application

Repeat application testing was defined as reapplication of specific oils to their respective disks at 3-day intervals over the course of the 21-day incubation period, resulting in a total of 7 applications per oil and organism for each experiment. A subset of essential oils (citronella, lemon grass, lemon myrtle, and litsea) and a single blend (On Guard) were selected based on demonstrated moderate activity in the initial screening assays. Moderate potency was defined as those individual oils with early zones of inhibition of 80 mm (complete inhibition) which subsequently collapsed in diameter >70% by the midpoint of the assay; or blends with a zone collapse of >50%. Repeat application was used to determine if the initial activity of each selected oil/blend tested could be maintained over the course of the 21-day assay vs. the zone collapse demonstrated in the initial screens. A single fungal isolate, *T. mentagrophytes* #2, was selected for the repeat application assays as this species/strain had the lowest average zone of inhibition for all essential oils considered together. Plates were monitored daily for growth and zones of inhibition measured as described above. Final zones of inhibition at day 21 of incubation were compared between repeat and single application and expressed as percent change by the assay endpoint at 21 days.

All assays as described above were performed in duplicate.

## Results

### Essential Oils Demonstrating Complete or Near Complete Inhibition

**Figure 1:**
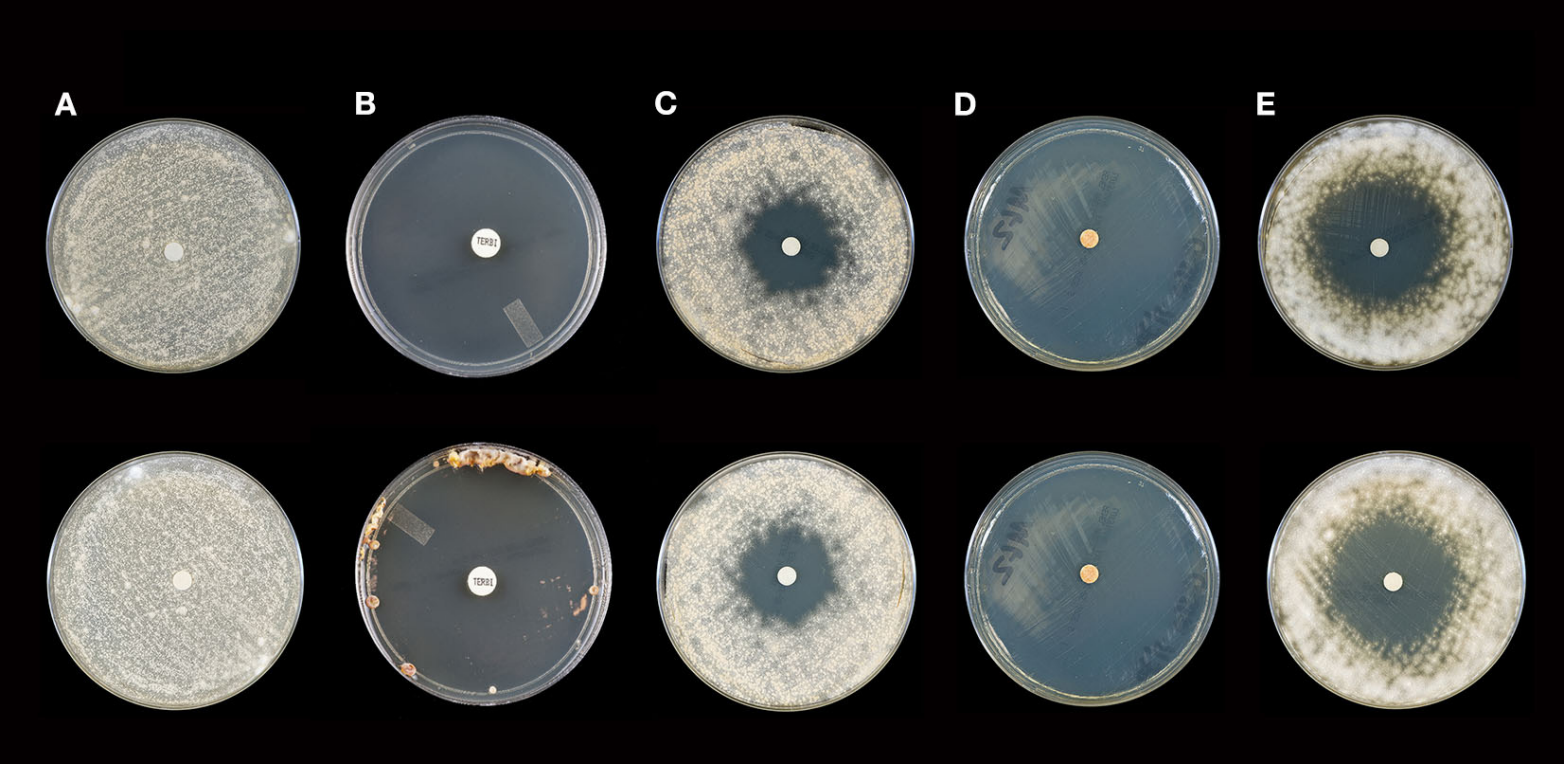
Activity of selected essential oils against *M. gypseum* strain #2 after 10 days of incubation (Top Panel) and 21 days of incubation (Bottom Panel). **(A)** Control [fractionated coconut oil): confluent lawn, no inhibition; **(B)** terbinafine (30 μg/ml): complete inhibition at 10 days, near complete inhibition (75 mm) at 21 days]; **(C)** itraconazole (10 μg/ml): little to no inhibition at 10 (30 mm) or 21 (28 mm) days; **(D)** cassia: complete inhibition at 10 and 21 days (80 mm), **(E)** arborvitae: moderate to minor inhibition at 10 (44 mm) and 21 (42 mm) days. Note: no marked changes in zone diameter between days 10 and 21.

**Figure 2:**
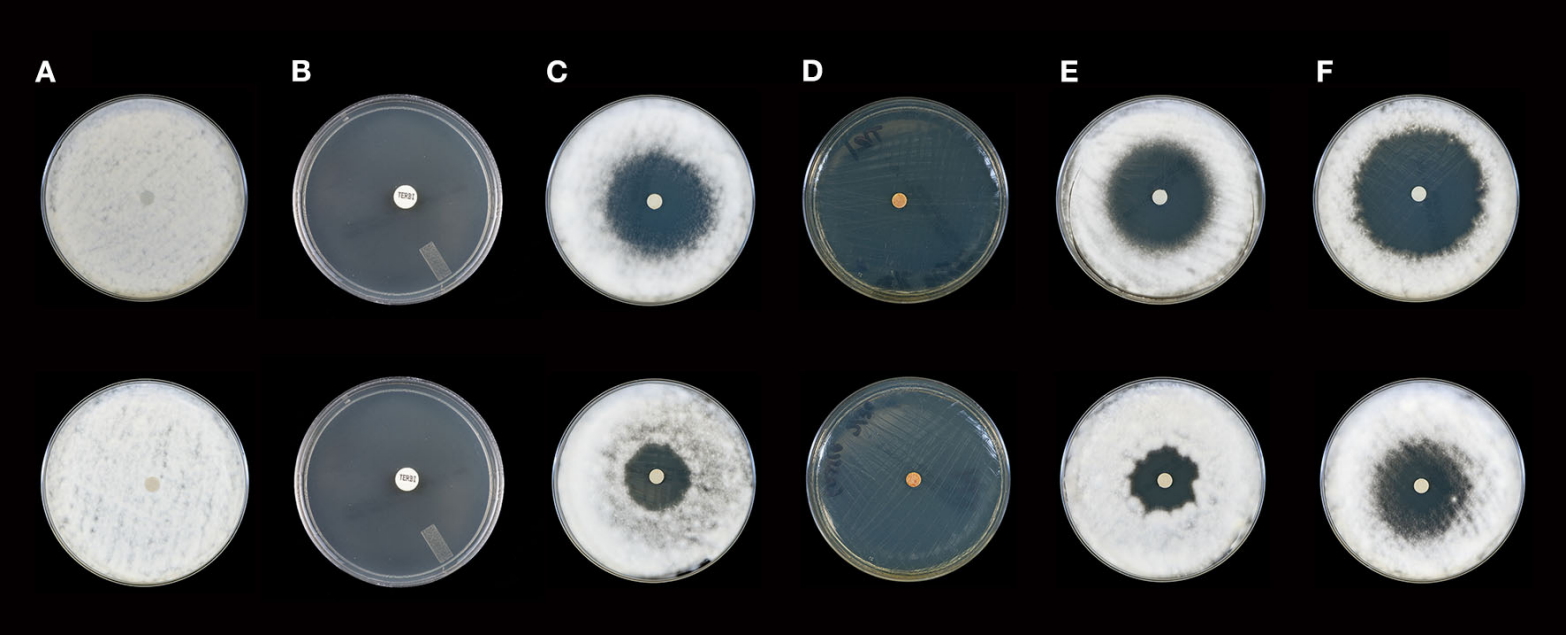
Activity of selected essential oils against *T. rubrum* strain #1 after 10 days of incubation (Top Panel) and 21 days of incubation (Bottom Panel). **(A)** Control (fractionated coconut oil): confluent lawn, no inhibition; **(B)** terbinafine (30 μg/ml): complete inhibition at 10 and 21 days (80 mm); **(C)** itraconazole, (10 μg/ml): minor to moderate inhibition at 10 days (36 mm), little to no inhibition at 21 days (23 mm); **(D)** cassia: complete inhibition at 10 and 21 days (80 mm), **(E)** bergamot: moderate to minor inhibition at 10 days (41 mm), little to no inhibition at 21 days (23 mm); **(F)** white fir: moderate to minor inhibition at 10 and 21 days with a decrease from 50 to 43 mm. Note: marked differences in zone diameter between days 10 and 21 in **(C,E,F)**.

Results for inhibitory effects of all essential oils are shown in Tables 2, 3; representative differences in inhibitory activity over time are illustrated in Figures 1, 2. The most potent single oils which showed complete inhibition following a single exposure over the entire length of the assay (21 days) include cassia, cilantro, oregano, thyme, and cinnamon along with one blend, DDR prime. For these oils, complete inhibition was demonstrated for all genera, species, and strains tested. No fungal growth was detected at any time point (indicated by a zone of inhibition equal to 80 mm or the diameter of the agar in the plate) during the assay following a single application of these respective oils and one blend. Many of the other oils and blends tested exhibited temporal differences in activity whereby potent early inhibition was observed (≤10 days of incubation) which later declined in varying degrees until assay completion at day 21 (Tables 2, 3). For example, litsea, rose, and lemon myrtle demonstrated complete inhibition of all growth initially for all genera, species, and strains tested. However, for these oils, fungal growth began to reappear by day 21 of the incubation period at the extreme margins of the agar plates for some species/strains such that measurable zones of inhibition could be determined (Table 3). The average zones of inhibition were still very large ranging from 79.4 mm for rose to 74.8 and 74.1 mm for litsea and lemon myrtle, respectively. Of note, fractionated coconut oil had no inhibitory effect on any of the fungal strains tested in this study. For terbinafine and itraconazole, the range for inhibition in mm at 21 days of incubation was established as 80 and 32 mm, respectively.

**Table 2:** Early activity (≤10 days) of single essential oils and blends as measured by zones of inhibition (mm).

**Single oils (*****n*** **=** **65)**				**Blends (*****n*** **=** **21)**	
**Essential oil**	**Average (range)**	**Essential oil**	**Average (range)**	**Essential oil**	**Average (range)**
Amyris	51.8 (0–80)	Howood	69.4 (0–80)	Anti-aging	53.2 (9–80)
Arborvitae	70.1 (45–80)	Jasmine	42.9 (17–80)	Aroma touch	73.7 (16–80)
Austrian fir	60.6 (24–80)	Juniper berry	26.6 (0–59)	Balancing	44.8 (0–80)
Basil	72.3 (31–80)	Labdanum	33.3 (0–80)	Breathe	67 (12–80)
Bergamot	38.5 (23–80)	Lavandin	73.3 (29–80)	Citrus bliss	46.3 (0–80)
Black cumin seed	32 (0–80)	Lavender	62.6 (0–80)	Clary calm	62.4 (9–80)
Black pepper	26.4 (14–41)	Lemon	18.4 (0–41)	Clear skin	62.3 (0–80)
Blue chamomile	47 (12–80)	Lemongrass	76.1 (17–80)	DDR prime	80 (80)
Blue tansy	55.4 (19–80)	Lemon myrtle	80 (80)	Deep blue	70.2 (15–80)
Camphor	71.1 (27–80)	Lime	28 (10–80)	Digestion	42.2 (12–80)
Cardamom	37.9 (19–80)	Litsea	80 (80)	Elevation	65.6 (16–80)
Cassia	80 (80)	Mandarin	45.1 (10–80)	Focus	49.6 (0–80)
Catnip	73.7 (51–80)	Marjoram	45.8 (19–80)	On guard	79.9 (78–80)
Cedarwood	24.7 (11–80)	Melaleuca	49.8 (0–80)	Purity	71.6 (9–80)
Cilantro	80 (80)	Myrrh	23.3 (10–80)	Serenity	64.8 (11–80)
Cinnamon	80 (80)	Oregano	80 (80)	Slim-n-Sassy	75.3 (46–80)
Citronella	79.1 (65–80)	Osmanthus (absolute)	78 (48–80)	Tension	74.9 (31–80)
Clary sage	45.5 (11–80)	Patchouli	57.3 (11–80)	Terra shield	35.3 (0–80)
Clementine	52.3 (16–80)	Peppermint	69.3 (29–80)	Topical	59.8 (0–80)
Clove	76.7 (65–80)	Roman chamomile	30.2(12–80)	Whisper	27.3 (0–80)
Cocoa	39.8 (0–80)	Rose	80 (80)	Zendocrine	74.4 (36–80)
Coriander	65.7 (0–80)	Rosemary	27.5 (0–80)		
Cypress	56 (10–80)	Sandalwood	29.9 (10–80)		
Eucalyptus	43.5 (12–80)	Siberian fir	68.8 (29–80)		
Fennel	36 (0–80)	Tangerine	49.4 (10–80)		
Fennel (sweet)	28.3 (11–80)	Thyme	80 (80)		
Fir needle	71.5 (37–80)	Vanilla	36.1 (0–80)		
Frankincense	17.8 (0–80)	Vetiver	31.7 (15–80)		
Geranium	76.7 (51–80)	White fir	34.9 (10–80)		
Ginger	78.1 (50–80)	Wild orange	19.9 (9–49)		
Grapefruit	22.7 (13–40)	Wintergreen	27.2 (0–80)		
H. Sandalwood	30.6 (0–80)	Ylang ylang	28.3 (10–57)		
Helichrysum	33.1 (12–80)				

**Table 3:** Late activity (21 days) of single essential oils and blends as measured by zones of inhibition (mm).

**Single oils (*****n*** **=** **65)**				**Blends (*****n*** **=** **21)**	
**Essential oil**	**Average (range)**	**Essential oil**	**Average (range)**	**Essential oil**	**Average (range)**
Amyris	43.1 (0–80)	Howood	49.5 (0–80)	Anti-aging	44.8 (9–80)
Arborvitae	49.3 (35–62)	Jasmine	29.1 (11–80)	Aroma touch	48.1 (0–80)
Austrian fir	48.7 (14–80)	Juniper berry	16.6 (0–39)	Balancing	24.8 (0–80)
Basil	20.9 (0–80)	Labdanum	12 (0–80)	Breathe	41 (0–80)
Bergamot	22.3 (12–32)	Lavandin	41.4 (0–80)	Citrus bliss	31.8 (0–80)
Black cumin seed	7.5 (0–80)	Lavender	13.4 (0–80)	Clary calm	37.7 (0–80)
Black pepper	16.4 (10–28)	Lemon	9.2 (0–20)	Clear skin	10.7 (0–52)
Blue chamomile	36.3 (12–80)	Lemongrass	80 (27–80)	DDR prime	80 (80)
Blue tansy	39.3 (9–80)	Lemon myrtle	74.1 (28–80)	Deep blue	27.1 (0–80)
Camphor	58.8 (16–80)	Lime	13.9 (0–30)	Digestion	6.9 (12–80)
Cardamom	19.3 (13–33)	Litsea	74.8 (27–80)	Elevation	47.3 (9–80)
Cassia	80 (80)	Mandarin	25.4 (0–80)	Focus	42.2 (0–80)
Catnip	58.9 (31–80)	Marjoram	14.9 (0–24)	On guard	75.1 (19–80)
Cedarwood	14.6 (10–22)	Melaleuca	15.8 (0–80)	Purity	52.6 (0–80)
Cilantro	80 (80)	Myrrh	13.7 (11–22)	Serenity	43.3 (0–80)
Cinnamon	80 (80)	Oregano	80 (80)	Slim-n-Sassy	60 (8–80)
Citronella	73.3 (15–80)	Osmanthus (absolute)	77.1 (36–80)	Tension	53.8 (0–80)
Clary sage	15.3 (0–31)	Patchouli	38.1 (12–80)	Terra Shield	11.1 (0–80)
Clementine	35.3 (0–80)	Peppermint	17.1 (0–80)	Topical	26.5 (0–80)
Clove	70.3 (0–80)	Roman chamomile	9.9 (0–18)	Whisper	16.3 (0–80)
Cocoa	19.9 (0–80)	Rose	79.4 (70–80)	Zendocrine	60.2 (0–80)
Coriander	14.4 (0–74)	Rosemary	1.1 (0–17)		
Cypress	39.7 (0–60)	Sandalwood	17.1 (8–27)		
Eucalyptus	6.1 (0–50)	Siberian fir	50.9 (15–80)		
Fennel	0.94 (0–15)	Tangerine	29.7 (0–80)		
Fennel (sweet)	10.7 (0–20)	Thyme	80 (80)		
Fir needle	59.5 (14–80)	Vanilla	18.1 (0–80)		
Frankincense	0 (0)	Vetiver	21.1 (12–32)		
Geranium	65.4 (24–80)	White fir	19.3 (0–45)		
Ginger	30.6 (0–80)	Wild orange	6.6 (0–18)		
Grapefruit	16.8 (10–30)	Wintergreen	2.2 (0–12)		
H. Sandalwood	18.3 (0–32)	Ylang ylang	14.6 (11–20)		
Helichrysum	17 (11–29)				

Other oils which demonstrated potent early activity, but slightly less than complete inhibition, included citronella, osmanthus, clove, geranium, ginger, lemon grass, and one blend, On Guard. Each of these oils/blend had average zones of inhibition in excess of 76 mm (Table 2, range 76.1–79.9 mm) during the initial incubation phase of the assay but at 21 days of incubation, zone diameters had decreased to an average of 66.9 mm (range 30.6–76.4 mm, Table 3). When compared to terbinafine, a standard antifungal commonly used for the treatment of dermatophyte infections, all of the oils/blends described thus far had comparable early activity against all species/strains tested with an average zone diameter of 79.5 mm (range 70.3–80 mm) vs. 74.5 mm for terbinafine. Importantly, by 21 days of incubation, the zone of inhibition for terbinafine had collapsed from 74.5 to 67.2 mm, whereas for the abovementioned oils/blends the average zone diameter at the same time point was 77.2 mm (range 70.3–80 mm). Itraconazole, another commonly used antifungal, showed only moderate inhibitory activity against all species/strains tested with early and late zone diameters of 40.2 and 23.3 mm, respectively.

### Essential Oils Demonstrating Moderate to Minor Inhibition

The majority of the remaining essential oils tested had early zones of inhibition which demonstrated moderate activity (Table 2, range 31.7–73.7 mm). However, for these oils/blends, many of these zones collapsed rapidly over the 21 days of incubation resulting in changes in zone diameters ranging from −17.3% for camphor and −82.8% for clear skin (Table 4). A few oils had little to no activity (Tables 3, 4). These oils [black pepper, fennel [sweet], grapefruit, H. sandalwood, juniper berry, lemon, lime, Roman chamomile, rosemary, sandalwood, wild orange, wintergreen, ylang ylang] and a single blend (Whisper) all had zones of inhibition <31 mm. Of these, the least potent was Frankincense which had an early average zone of inhibition of 17.8 mm which completely collapsed by day 21 where fungal growth grew in a confluent lawn up to the disk.

**Table 4:** Comparative activity of single oils at 21 days of incubation by genus, species, and strain.

**Isolate**	**Terbinafine**	**ITRA**	**Amyris**	**Arborvitae**	**Austrian fir**	**Basil**	**Bergamot**	**Black cumin seed**	**Black pepper**	**Blue chamomile**	**Blue tansy**	**Camphor**	**Cardamom**	**Cassia**	**Catnip**	**Cedarwood**	**Cilantro**	**Cinnamon**	**Citronella**	**Clary sage**	**Clementine**	**Clove**	**Cocoa**	**Coriander**	**Cypress**	**Eucalyptus**	**Fennel**	**Fennel (sweet)**	**Fir needle**	**Frankincense**	**Geranium**	**Ginger**	**Grapefruit**	**H. Sandalwood**	**Helichrysum**	**Howood**	**Jasmine**	**Juniper berry**	**Labdanum**	**Lavandin**	**Lavender**	**Lemon**	**Lemon grass**	**Lemon myrtle**	**Lime**	**Litsea**	**Mandarin**	**Marjoram**	**Melaleuca**	**Myrrh**	**Oregano**	**Osmanthus (absolute)**	**Patchouli**	**Peppermint**	**Roman chamomile**	**Rose**	**Rosemary**	**Sandalwood**	**Siberian fir**	**Tangerine**	**Thyme**	**Vanilla**	**Vetiver**	**White fir**	**Wild orange**	**Wintergreen**	**Ylang ylang**	**Average ZOI (mm)**
TT 1	30	0	35	34	43	9	17	0	13	33	21	46	14	80	80	10	80	80	40	9	24	0	0	0	34	0	0	9	42	0	24	0	13	14	11	80	23	18	9	19	0	11	80	80	11	80	14	11	0	12	80	80	22	16	10	80	0	17	38	21	80	10	18	0	0	0	11	26.50746
TT 2	63	23	27	50	48	16	18	0	14	23	21	44	15	80	47	22	80	80	80	17	25	80	0	0	36	50	0	12	45	0	80	24	15	20	19	32	26	20	10	20	23	10	80	80	10	80	17	19	59	22	80	78	35	17	0	80	0	22	42	17	80	9	32	26	0	0	20	33.13433
TV 1	80	24	36	57	61	80	31	0	27	29	26	59	33	80	80	21	80	80	78	31	42	80	15	74	55	0	15	16	72	0	80	21	25	29	20	51	26	17	12	42	46	20	80	80	20	80	11	18	40	16	80	80	80	0	18	80	17	26	42	29	80	12	25	13	18	12	12	40.59701
TV 2	80	32	69	62	60	20	21	30	14	53	48	80	20	80	77	16	80	80	80	20	45	80	45	17	47	0	0	16	80	0	80	80	22	31	15	57	48	33	44	51	29	13	80	80	0	80	53	18	0	14	80	80	42	20	13	80	0	21	73	50	80	17	30	45	14	0	20	42.46269
TV 3	80	25	58	41	66	12	26	0	16	29	25	60	21	80	80	21	80	80	80	14	46	80	17	12	51	0	0	11	67	17	80	45	20	18	16	39	29	0	14	26	0	15	80	70	17	80	24	19	16	16	80	80	51	31	15	80	0	28	49	31	80	11	25	37	11	9	18	36.64179
TS 1	80	21	80	47	63	0	12	0	10	80	80	80	13	80	80	18	80	80	80	0	46	80	0	0	28	0	0	10	80	0	43	42	10	0	13	80	17	0	23	80	22	0	80	80	11	80	36	0	19	18	80	80	40	14	0	80	0	15	80	37	80	0	29	25	0	0	11	36.61194
TS 2	80	0	80	58	37	11	15	0	con	80	80	68	15	80	51	18	80	80	80	0	35	80	13	0	35	29	0	0	80	0	80	29	16	32	19	9	15	21	0	80	80	11	80	80	11	80	16	10	24	18	80	80	80	0	0	80	0	C	51	30	80	0	C	0	C	C	C	38.96721
TR 1	65	0	9	50	46	17	22	0	13	16	15	55	18	80	31	14	80	80	80	13	24	80	8	0	0	0	0	12	50	0	80	31	13	14	16	80	15	0	0	28	0	10	80	80	30	80	7	17	0	11	80	80	46	0	14	80	0	11	46	20	80	8	22	36	0	0	10	29.89552
TR 2	71	20	24	50	23	18	23	10	13	16	14	63	18	80	42	12	80	80	80	14	49	80	13	22	43	0	0	9	52	0	80	35	15	18	14	44	14	0	0	33	0	0	80	80	16	75	55	0	0	11	80	80	28	32	9	80	0	8	51	41	80	14	12	33	0	0	10	32.04478
TM 1	69	8	18	43	47	19	21	0	15	13	15	56	15	80	33	10	80	80	80	15	24	60	8	20	40	0	0	12	50	0	73	0	12	11	13	80	15	22	0	23	0	0	80	80	11	80	10	19	0	10	80	80	12	0	10	80	0	10	46	20	80	8	15	8	0	0	13	28.83582
TM 2	49	14	0	45	14	10	23	0	16	12	9	16	17	80	32	10	80	80	15	12	0	80	0	0	42	0	0	10	14	0	73	53	16	16	29	0	11	26	0	0	0	13	80	28	16	27	0	15	0	12	80	36	16	0	14	80	0	14	15	0	80	0	18	8	12	0	16	22.14925
MG 1	60	13	17	43	38	23	19	0	12	16	19	38	21	80	33	10	80	80	80	19	20	50	0	18	23	0	0	0	43	0	37	15	12	12	19	0	17	8	0	17	0	10	80	80	12	80	9	20	0	10	80	80	34	0	9	80	0	11	22	10	80	0	12	31	8	10	11	26.43284
MG 2	66	17	17	42	27	19	17	0	12	16	15	36	15	80	36	10	80	80	80	17	26	55	0	13	38	0	0	0	37	0	34	0	13	16	11	0	15	18	0	9	0	11	72	48	12	54	15	16	0	10	80	80	26	0	9	80	0	10	27	16	80	0	13	0	0	0	13	24.46269
MC 1	70	30	59	58	80	34	31	0	23	14	80	80	22	80	80	15	80	80	80	27	31	80	79	20	53	0	0	20	80	0	80	0	30	20	22	80	51	39	0	80	0	0	80	80	12	80	14	24	0	11	80	80	36	0	15	70	0	27	80	28	80	80	24	14	16	0	19	40.86567
MC 2	80	34	80	56	80	27	32	80	28	80	80	80	26	80	80	14	80	80	80	19	80	80	80	21	60	0	0	19	80	0	42	80	ND	ND	ND	80	63	ND	80	80	0	ND	ND	80	ND	80	80	ND	0	ND	80	80	ND	64	ND	80	0	ND	80	80	80	80	ND	ND	ND	ND	ND	58.1
MA 1	80	22	80	60	46	19	28	0	20	71	80	80	26	80	80	13	80	80	80	18	48	80	41	14	50	0	0	15	80	0	80	24	20	24	18	80	80	27	0	75	14	14	80	80	19	80	45	18	0	15	80	80	24	80	13	80	0	19	73	45	80	40	21	13	14	0	20	42.47761

*ND, not done; C, contaminated; ZOI, zone of inhibition in mm*.

### Differences in Essential Oil Activity Stratified by Genera, Species, and Strain

Differences in essential oil activity were noted between genera, species, and strains. Overall, when considering the total number of results demonstrating complete inhibition at 21 days of incubation for all species and strains within a genus, *Microsporum* showed 28.6% or 91/318 total tests resulting in zone diameters of 80 mm vs. *Trichophyton* for which 20.1% (152/734) was observed. One strain of *M. canis* (MC #2) tested was the most susceptible to the majority of the oils tested consistently demonstrating large zones of inhibition with an average of 58.1 mm (range 0–80 mm) (Tables 4, 5); whereas, *T. mentagrophytes* (strain #2) was the most resistant with an average zone of inhibition of 22.1 mm (range 0–80 mm). Strain-to-strain differences were also noted within the same species (Tables 4, 5). For example, the two strains of *T. tonsurans* tested demonstrated differential susceptibility to clove and eucalyptus where strain 2 showed zones of 80 and 50 mm, respectively, vs. no activity for the same oils against strain 1. Likewise, the same was noted for several other species/strains including the *M. canis* strains tested where no activity was noted for ginger and peppermint (strain 1) vs. zones of 80 and 64 mm, respectively, for strain 2.

**Table 5:** Comparative activity of Blends by genus, species and strain at 21 days of incubation.

**Isolate**	**Anti-aging**	**Aroma touch**	**Balancing**	**Breathe**	**Citrus bliss**	**Clary calm**	**Clear skin**	**DDR prime**	**Deep blue**	**Digestion**	**Elevation**	**Focus**	**On guard**	**Purity**	**Serenity**	**Slim/ sassy**	**Tension**	**Terra shield**	**Topical**	**Whisper**	**Zendocrine**
TT 1	25	23	10	0	8	28	0	80	29	0	36	24	80	55	23	80	63	0	27	10	63
TT 4	22	24	11	27	8	29	80	80	80	21	35	23	80	62	22	47	33	0	16	11	75
TV 1	36	39	19	34	39	34	52	80	69	0	48	36	80	78	39	75	65	8	19	13	80
TV 2	80	70	38	66	48	34	32	80	0	0	80	65	80	80	80	80	80	31	48	50	80
TV 3	33	43	19	36	12	36	71	80	13	0	43	32	80	80	33	75	65	11	19	10	80
TS 1	80	80	19	80	22	80	26	80	66	61	80	80	80	0	80	80	28	12	0	11	0
TS 2	80	80	11	31	11	37	25	80	80	0	80	80	80	17	80	80	20	0	0	9	80
TR 1	14	31	13	27	25	27	80	80	80	0	27	17	62	34	28	49	80	0	18	9	68
TR 2	36	34	19	33	29	35	0	80	0	0	39	27	80	80	25	36	61	11	23	13	80
TM 1	20	27	14	30	25	24	0	80	0	0	27	18	0	68	11	42	80	0	25	10	46
TM 3	9	0	0	0	0	0	0	75	18	0	9	0	80	0	0	8	0	0	0	0	15
MG 1	16	10	0	22	6	15	0	80	0	0	18	20	19	35	14	36	23	0	0	8	36
MG 2	80	68	80	80	80	21	0	80	0	0	28	13	0	12	17	32	23	0	0	0	23
MC 1	40	80	32	30	80	43	0	80	0	0	80	80	80	80	80	80	80	0	80	8	80
MC 2	80	80	80	80	80	80	0	80	0	0	47	80	80	80	80	80	80	80	80	80	80
MA 2	66	80	32	80	35	80	14	80	12	0	80	80	80	80	80	80	80	25	69	19	80

### Minimum Inhibitory Concentrations (MIC's) for the Most Potent Essential Oils

Table 6 illustrates the lowest 2-fold dilution of each of the most potent essential oils as determined in the initial screens which resulted in complete inhibition of the selected strains tested. As shown, cassia demonstrated the most potent activity with MICs of <1:8 (v/v) for 7 of the 11 (63.6%) species/strains tested. Similar results were obtained for cinnamon bark where 54.5% (6/11) of the species/strains tested had MICs equivalent to a dilution of 1:8 (v/v). Overall, cassia, cinnamon bark, and oregano had MICs ≤ to 1:4 (v/v) for 90.9% (10/11) of the species/strains tested. Cilantro, citronella, and thyme demonstrated more moderate potency where the number of species/strains with MICs ≤1:4 (v/v) was 63.6% (7/11), 72.7% (8/11), and 72.7% (8/11), respectively. Of the other oils tested (rose, osmanthus, lemon myrtle, litsea, and lemon grass), most had to be used neat for complete inhibition (Table 6).

**Table 6:** Minimum inhibitory concentrations of selected essential oils with the highest activity for all genera, species and strains considered together.

**Isolate**	**Essential oil**										
	**Cassia**	**Cinnamon Bark**	**Cilantro**	**Citronella**	**Lemon Grass**	**Lemon Myrtle**	**Litsea**	**Oregano**	**Osmanthus (absolute)**	**Rose**	**Thyme**
TT 1	<1:8	1:8	1:8	1:4	1X	1X	1X	1:4	1:2	1X	1:4
TT 2	1:4	1:8	1:4	1:4	1x	1:2	1:2	1:4	1:2	1x	1:4
TV 1	<1:8	1:4	<1:8	1:2	1:4	1X	1X	<1:8	1:2	1X	1:4
TV 2	<1:8	1:4	1:4	1:2	1:2	1:2	1:4	1:4	1:2	1X	1:4
TR 1	1:4	1:8	1:2	1:4	1X	1X	1X	1:4	1X	1X	1:2
TR 2	<1:8	1:8	1X	1:4	1:4	1X	1:4	1:8	1:2	1X	1:8
TM 1	1:2	1:2	1:2	1X	1:4	1X	1:2	1:4	1X	1X	1:4
TM 2	1:4	1:4	1:2	1:4	1X	1X	1:2	1:4	1X	1X	1:4
MG 1	<1:8	1:4	1:4	1:4	1X	1X	1X	1:4	1X	1X	1:2
MG 2	<1:8	1:8	1:4	1:4	1X	1X	1X	1:4	1X	1X	1:2
MC 1	<1:8	1:8	1:4	<1:8	1X	1:4	1X	1:2	1:4	<1:8	1:4

*Note: For results of 1X, the essential oils had to be used “neat” (undiluted)*.

### Synergy Testing

Sub-inhibitory concentrations of selected individual oils used in combination resulted in synergy for the four species/strains selected for testing. Synergy was defined as combinations of diluted essential oils which resulted in complete zones of inhibition of 80 mm at day 21 of incubation vs. development of a complete lawn with the same concentration of each individual essential oil alone. Results are shown in Table 7. Combinations with oregano (1:8, v/v), were synergistic with cilantro (1:8, v/v) against *T. tonsurans* #2, *T. rubrum* #1, and *M. gypseum* #1. Oregano (1:8 v/v) was also synergistic with cassia (1:8, v/v) for *T. rubrum* #1 and *T. mentagraphytes* #1. Interestingly, for one strain, *T. tonsurans* #2, the cassia dilution in combination with oregano required for complete inhibition at 21 days was 1:32 (v/v) vs. 1:8 (v/v) observed for the other strains/combinations. Oregano (1:8, v/v) was also paired with cinnamon bark (1:16, v/v) resulting in complete inhibition at 21 days for *T. tonsurans* #2 and *M. gypseum* #1. Rose (1:2, v/v) and cassia (1:8, v/v) were found to completely inhibit *T. mentagraphytes* #1 at 21 days of incubation.

**Table 7:** Synergistic combinations of sub-inhibitory concentrations of various essential oils combinations against selected dermatophyte strains at 21 days of incubation.

**Essential oil combinations**	**Isolate**			
	***T. tonsurans* #2**	***T. rubrum* #1**	***M. gypseum* #1**	***T. mentagrophytes* #1**
Oregano (1:8, v/v) + Cilantro (1:8, v/v)	+	+	+	ND
Oregano (1:8, v/v) + Cassia (1:8, v/v)	ND	+	ND	+
Oregano (1:8, v/v) + Cassia (1:32, v/v)	+	ND	ND	ND
Oregano (1:8, v/v) + Cinnamon Bark (1:16, v/v)	+	ND	+	ND
Rose (1:2, v/v) + Cassia (1:8, v/v)	ND	ND	ND	+

“*+”, Synergistic; “ND”, Not done*.

### Repeat Application

Repeat dosing at 3-day intervals for citronella, lemon myrtle, and litsea resulted in maintenance of complete growth inhibition (zone diameter = 80 mm) to the assay endpoint of 21 days. This is in contrast to the single application of the same oils where the zone collapse at 21 days of incubation was −82.5% for citronella (80 mm down to 14 mm), 73.8% for lemon myrtle (80 mm down to 21 mm), and 71.3% for litsea (80 mm down to 23 mm). Repeat application with lemon grass resulted in a change in zone collapse from −73.8% (80 mm down to 21 mm) to −45.0% (80 mm down to 44 mm). However, unlike the other three oils mentioned above, repeat application did not maintain complete inhibition (80 mm) to the end of the study (21 days). On Guard, the only blend tested, showed no improvement with repeated application with a final zone diameter of 45 mm in both assays.

## Discussion

The essential oils used in this study were diverse in composition and selected to provide a wide variety for comparative activity studies against a number of clinically important dermatophytes. Our study was comprehensive and applied systematic rigor of testing following established laboratory procedures for antimicrobial/antifungal susceptibility testing. This approach was chosen to provide robust and reproducible data for further interpretation. Furthermore, such a comprehensive study approach was envisioned to provide more of a sense as to whether or not the antifungal activity was generalizable to a wide variety of oils or if there were differences in activity between them which could then be investigated further with regard to their respective components. In so doing, we noted distinct differences in antifungal activity between various essential oils where some had potent activity and some did not. For instance, some oils which demonstrated little to no activity such as lemon, lime, wild orange, tangerine, and mandarin are all known to contain limonene as a primary component. Some also contain α-pinene or ⋎-terpinene as one of the top 3 components in varying ratios. Despite these similarities in composition, clear differences in activity were observed which were not easily explained. In fact, frankincense demonstrated the least amount of inhibition of any of the essential oils tested yet is known to have limonene as a primary constituent. Clearly, other compounds or components in lesser abundance may contribute to the inhibitory activity observed in this study. This is also in contrast to what Chee and coworkers observed in their study (2009) in which limonene alone in concentrations of 0.5% v/v inhibited the dermatophyte *T. rubrum* in a broth microdilution assay (Chee and Lee, 2009). It is possible that since the limonene contained in our essential oils was diluted with other components and a carrier (fractionated coconut oil), that the overall concentration of limonene was less than that utilized by the prior investigators, resulting in decreased inhibitory activity as observed in our study. Perhaps also of relevance is the fact that the current study utilized a modified disk-diffusion assay whereby not only contact inhibition was possible by essential oils diffusing through the agar, but also volatile compounds contained within the sealed plates. This may explain some of the differences in the observed activity of individual essential oils used in our study vs. prior investigations. However, previous investigators also demonstrated that primary components such as linalool in concentrations as low as 0.09–0.29% were inhibitory for some clinically relevant fungi, whereas in the current study, howood, containing 98% linalool by abundance showed only moderate inhibition against all genera, species, and strains tested with an average zone of 49.5 mm. In contrast, cilantro which contained a lesser amount of linalool (35% by abundance) clearly demonstrated more inhibitory activity over the 21 days of the assay suggesting once again that other minor components within the essential oil alone or in combination are responsible for the antifungal activity observed in this study. The current study was limited by the fact that we did not test or evaluate the exact content of the individual components of each oil. Such additional testing to determine the impact of each individual component and combination of components was beyond the scope of this initial survey. This study was also limited in the number of clinical species and strains tested and did not include strains of *Epidermophyton*. Thus, essential oil-mediated inhibition against the dermatophytes observed in this study may be less accurate with some genera/species. This is especially possible with regard to *M. canis* where, although initial testing was completed with all of the oils for strains #1 and #2, the loss of viability in strain #2 prevented any further testing including determination of individual MICs or synergies. Interestingly, *M. canis* strain #2 exhibited very slow growth throughout the initial testing phase which may have contributed to the markedly increased zones of inhibition observed for most oils relative to *M. canis* strain #1. This same slow growth may have contributed to the eventual loss in viability as well. As a result of this loss, comprehensive results were not available for this particular genus/species and testing is warranted with additional *M. canis* strains.

Previous investigators, working largely with individual essential oils, suggested that the mechanism of action of these complex mixtures involves primarily alteration of cellular permeability (Flores et al., 2015). However, other studies have postulated that synergism between components, including minor ones by abundance, may lead to a complex mechanism of action which is multifactorial. In our study, the picture is even more complex, as differences in activity were noted between different genera, species, and strains. If disruption of the cellular membrane is the primary mode of action for all essential oils, then a more generalizable effect would be expected, especially with closely related organisms such as the ones tested in the current study. However, that was not the case; in fact some oils which displayed strong potency against one strain of a given genus and species (clove against TT2) had little to no effect on another strain of the same species (clove against TT1). In addition, synergy was demonstrated with combinations of essential oils selected from different groups based on the predominant constituent(s) known to be present such as aldehydes, phenols, and alcohols. The differential activity between various genera, species and strains as well as the synergy between chemically different essential oils suggests that other strain or species-specific intrinsic factors involving a yet unknown mechanism(s) may be responsible for the previously and currently observed variations in activity.

For some oils, the inhibition demonstrated in this study was not unexpected. Previous investigators had shown that essential oils such as oregano, thyme, and clove inhibited various yeasts such as *Sacharomyces cerevesiae* and *Candida* spp. (Dias de Castro et al., 2015). Essential oil mediated inhibition was postulated to be due to the binding of particular components (thymol) of some essential oils to ergosterol which affects membrane permeability resulting in inhibition of hyphal growth and conidia production. Carvacrol, a phenolic, monoterpene derivative of cymene, is commonly found in oregano essential oil, and can interrupt the cell cycle in eukaryotic cells as well as disrupt and depolarize the plasma membrane (Dai et al., 2016). It has also been postulated to have an inhibitory effect on endoplasmic reticulum and protein synthesis. Still other components such as cinnamaldehyde found in cassia and cinnamon bark are known to negatively affect spore production in *Aspergillus flavus* (Sun et al., 2016).

## Conclusion

Since antiquity and even today in popular culture, essential oils have been used to treat a number of maladies including fungal infections of the skin, hair, and nails. Although EOs are utilized globally as an adjunct to alternative medicine including aromatherapy, their use in mainstream medicine as antimicrobials has yet to happen. Currently, the number of treatment refractory dermatophyte infections are increasing and at the time of writing this manuscript, FDA approved treatments are limited (Ghannoum et al., 2000; Santos and Hamdan, 2007; Gupta et al., 2017). Development of new antifungal agents is warranted as older antifungals may become less relevant over time and are made irrelevant by the emergence of resistance the latter of which highlights the importance of developing novel and alternate treatments. However, additional studies will be necessary to provide a more comprehensive understanding of the mechanism of action of specific essential oils against individual fungi of clinical importance. Our current understanding of the mechanism of action of these complex mixtures is rudimentary at best as the intricacies of the interplay between predominant and lesser components of individual oils is seriously lacking. To date, no studies have been performed which investigate all of the components for a given oil in any kind of comprehensive way. Thus, it is not known to any extent what contribution lesser components provide to the antimicrobial activity observed in other studies as well as our own. Future studies using animal models may be helpful in understanding the efficacy of EOs against dermatophytes *in vivo*. It is also likely that novel drug targets may be discovered as a result of this process leading to new scaffolds for drug development.

## Data Availability Statement

All datasets generated in this study are included in the article/ supplementary material.

## Author Contributions

NP, DC, JK, SR, and SZ contributed to the writing of the manuscript. NP, SR, and SZ planned experiments. SF, AG, NB, and JK conducted laboratory experiments. All authors contributed to the article and approved the submitted version.

## Conflict of Interest

The authors declare that the research was conducted in the absence of any commercial or financial relationships that could be construed as a potential conflict of interest.
